# SNHG16/miR-140-5p axis promotes esophagus cancer cell proliferation, migration and EMT formation through regulating ZEB1

**DOI:** 10.18632/oncotarget.23178

**Published:** 2017-12-11

**Authors:** Kai Zhang, Jing Chen, Haizhu Song, Long-Bang Chen

**Affiliations:** ^1^ Department of Medical Oncology, Jinling Hospital, School of Medicine, Nanjing University, Nanjing, Jiangsu, China

**Keywords:** esophagus cancer, long non-coding RNA, SNHG16, proliferation, EMT

## Abstract

Esophageal squamous cell carcinoma (ESCC) is one of the most aggressive malignancies. Long noncoding RNAs (lncRNAs) have been identified to be associated with many diseases including tumors, and involved in the regulation of a wide array of pathophysiological processes. Small nucleolar RNA host gene 16 (SNHG16), also known as noncoding RNA expressed in aggressive neuroblastoma, was newly identified as a potential oncogene in many cancers. However, its role in ESCC has not been investigated. In the current study, the level of SNHG16 in the ESCC tissues and cell lines was measured by quantitative real-time PCR (qRT-PCR). Then loss-of-function assays were performed to explore the biological effects of SNHG16 in ESCC cell. Based on the online database analysis tools, we uncovered that miR-140-5p could interact with SNHG16 and the level of miR-140-5p was inverse correlated with SNHG16 in ESCC specimens. Moreover, RIP, RNA pulldown system and dual luciferase reporter assay further provided evidence that SNHG16 directly targets miR-140-5p by binding with microRNA binding site harboring in the SNHG16 sequence. Furthermore, bioinformatics analysis revealed that ZEB1 is a target of miR-140-5p in ESCC. Collectively, our findings suggested that SNHG16 could act as an oncogenic lncRNA that promotes tumor progression through acting as an endogenous ‘sponge’ by competing with miR-140-5p, thereby regulating target ZEB1.

## INTRODUCTION

Esophageal cancer (EC) is one of the most common types of digestive tract cancer [1]. About 90% of EC cases in China are esophageal squamous cell carcinoma (ESCC), which is characterized by poor prognosis and high mortality rate [2]. Due to diagnose at advanced stage and lacking effective treatment targets, the prognosis of ESCC still remains poor. ESCC is a multistep process involving a series of genetic or epigenetic alterations [3–5]; thus, it is essential to investigate the molecular and pathogenic mechanisms underlying ESCC carcinogenesis which might be helpful for improving diagnosis and management of human ESCC.

It has been demonstrated that the majority (∼97%) of human genome sequences are transcribed into noncoding RNAs (ncRNAs). Based on the length, these ncRNAs could be divided into two groups, including short ncRNAs (<200 nt) and long ncRNAs (>200 nt) [6, 7]. Among the short ncRNAs, microRNAs are the major member, which have been been demonstrated to play critical roles in ESCC development. For instance, antagonizing miR-455-3p inhibits chemoresistance and aggressiveness in esophageal squamous cell carcinoma [8]. LncRNAs, as novel proposed ncRNAs, have been reported to be involved in multiple cancers [9–12], including ESCC. For example, transcriptional and posttranscriptional regulation of HOXA13 by lncRNA HOTTIP facilitates tumorigenesis and metastasis in esophageal squamous carcinoma cells [13].

Currently, a novel post-transcriptional regulation model was proposed that lncRNAs containing miRNA response elements (MREs) function as competitive endogenous RNAs (ceRNAs) competitively sponging target miRNAs through binding to MREs [14–20]. Small nucleolar RNA host gene 16 (SNHG16) was newly identified as a potential oncogene in lung cancer, breast cancer, bladder cancer and colorectal cancer [21–24]. However, the biological role and potential mechanism of SNHG16 in ESCC remains unclear.

The present study aimed to comprehensively explore the biologic functions and underlying mechanisms of SNHG16 in ESCC cells. Our findings presented that SNHG16 is upregulated in ESCC tissues and cells and is involved in the proliferation and migration of ESCC via regulating miR-140-5p/ZEB1 signal pathway.

## RESULTS

### SNHG16 was up-regulated in ESCC tissues and cell lines

**Figure 1:**
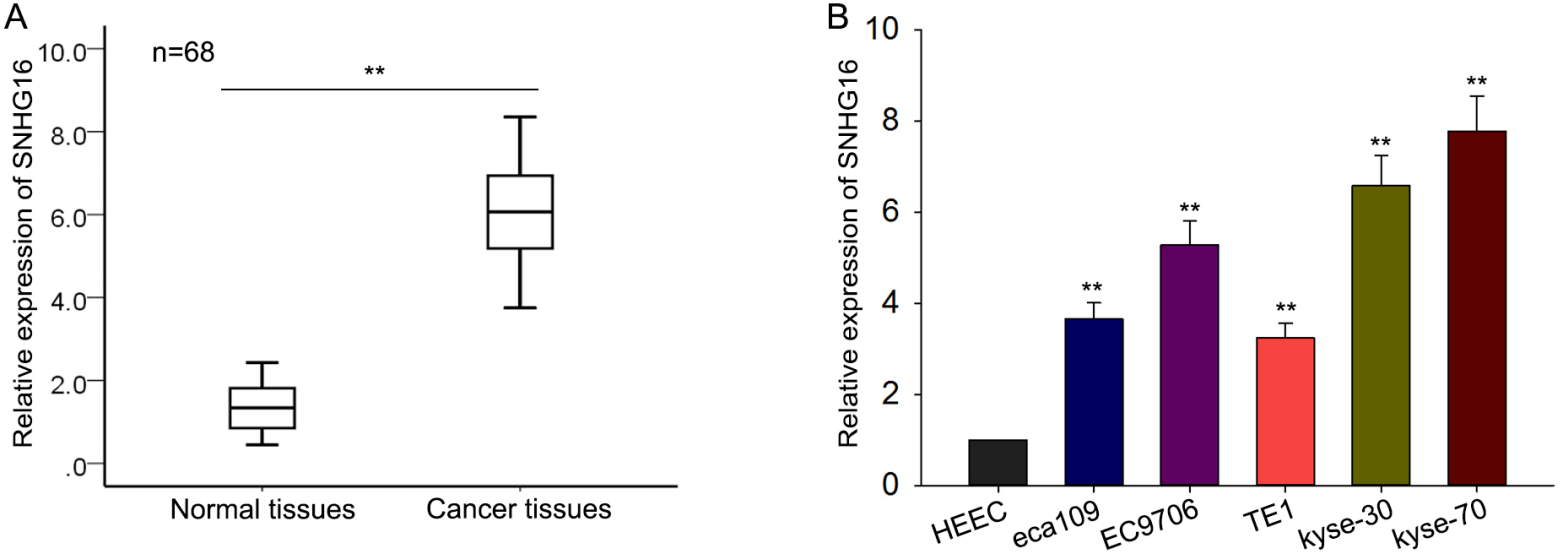
SNHG16 is up-regulated in ESCC tissues and cell lines **(A-B)** qRT-PCR was performed to measure the level of SNHG16 in ESCC tissues and cell lines. Error bars represent the mean ± SD of at least three independent experiments. ^*^p<0.05, ^**^p<0.01 vs. control group.

To investigate the expression pattern, biological function and underlying mechanism of SNHG16 in the tumorigenesis of ESCC, we first measured the SNHG16 expression level in 68 pairs of ESCC tissues and corresponding histologically normal tissues by qRT-PCR. As shown in Figure 1A, the level of SNHG16 in ESCC tissues was significantly increased, compared with matched noncancerous tissue. To make further confirmation, we then assessed the expression level of SNHG16 in five ESCC cell lines (eca109, EC9706, TE1, kyse-30 and kyse-70) and a normal esophageal epithelial cell line (HEEC). The results revealed an increased SNHG16 expression in ESCC cells compared with HEEC cells (Figure 1B). Based on the data referred above, we suspected that SNHG16 may be involved in the progression of the ESCC.

### Silencing SNHG16 suppressed ESCC cells proliferation and induced apoptosis

**Figure 2:**
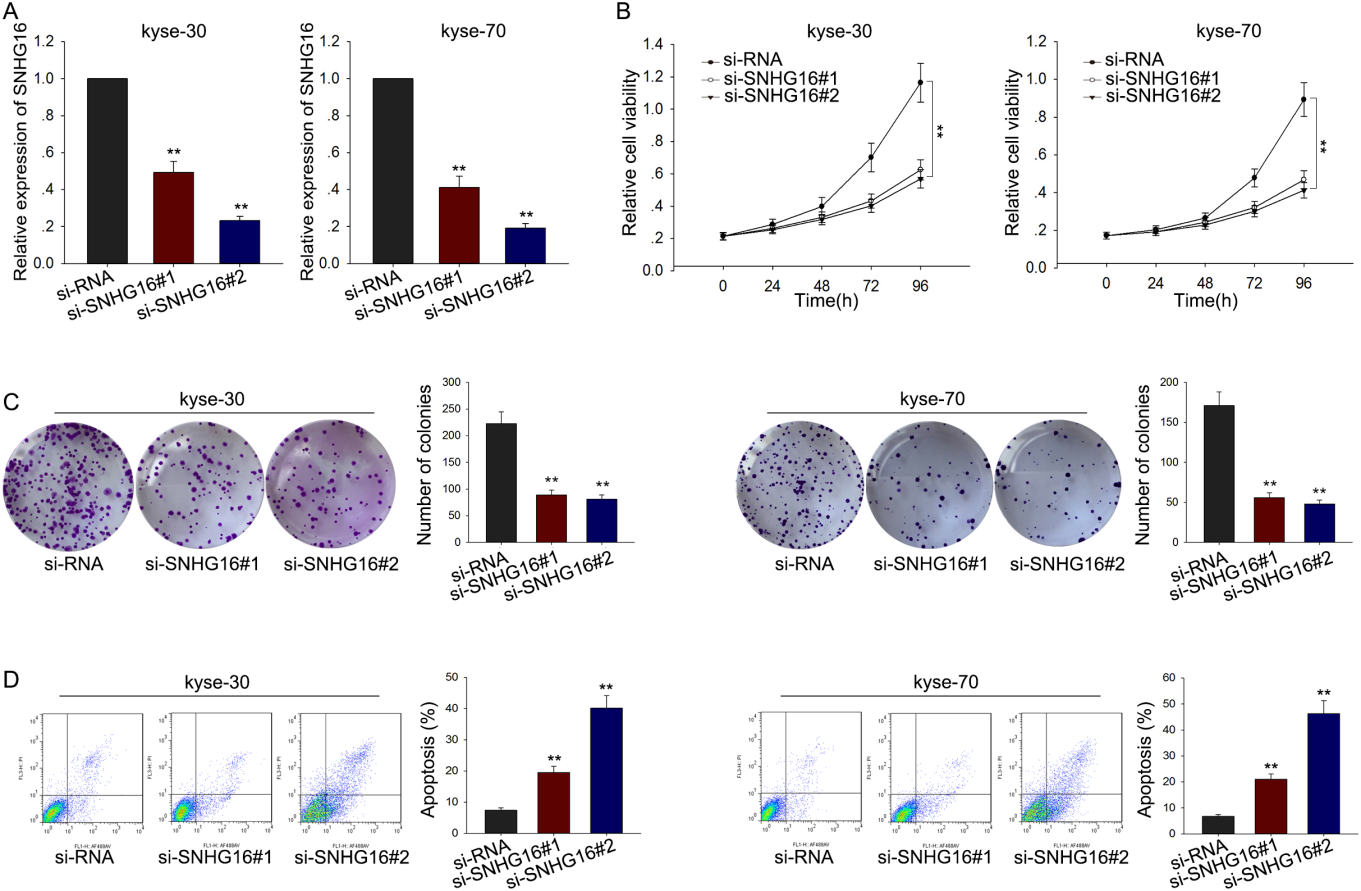
Silencing SNHG16 suppressed ESCC cells proliferation and induced apoptosis **(A)** qRT-PCR was performed to detect the transfection efficiency of SNHG16 specific siRNA. **(B-C)** MTT and colony formation assay were performed to detect the proliferation ability of ESCC cells transfected with si-SNHG16. **(D)** Flow cytometric analysis was performed to determine the effect of SNHG16 on cell apotosis. Error bars represent the mean ± SD of at least three independent experiments. ^*^p<0.05, ^**^p<0.01 vs. control group.

To determine the biological function of SNHG16, SNHG16 specific siRNA was used to silence the level of SNHG16 in kyse-30 and kyse-70 cells, using the si-RNA as a negative control (NC). Satisfactory transfection efficiency was obtained at 48 h post-transfection (Figure 2A). Then, MTT assay was performed to detect the proliferation ability of ESCC cells transfected with si-SNHG16. As illustrated in Figure 2B, results from MTT assay showed that weakened proliferation ability was observed in ESCC cells transfected with si-SNHG16, compared with NC-transfected cells. Consistently, results from colony formation assay further confirmed the decreased proliferation ability of ESCC cells transfected with si-SNHG16 (Figure 2C). To determine the effect of SNHG16 on cell apoptosis, flow cytometric analysis was performed. As demonstrated in Figure 2D, silenced SNHG16 obviously increased the apoptosis rate of ESCC cells, in comparison to negative control cells. These data together demonstrated that SNHG16 was involved in the proliferation and apotpsis of ESCC cells.

### Knockdown of SNHG16 inhibited ESCC cells migration through blocking the EMT phenotype formation

**Figure 3:**
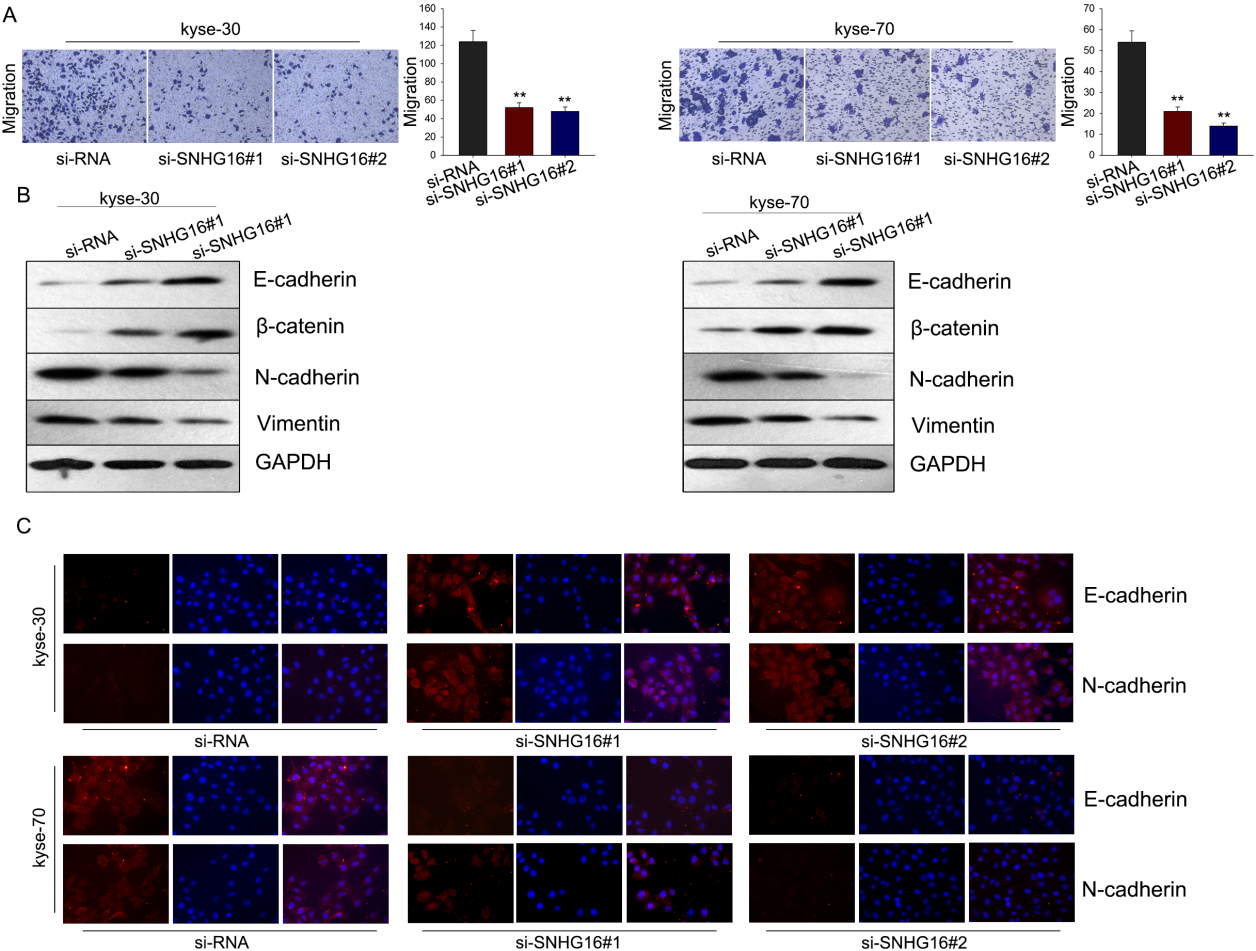
Knockdown SNHG16 inhibited ESCC cells migration through blocking the EMT phenotype formation **(A)** Transwell assay was employed to evaluate the effect of SNHG16 on cell migration. **(B-C)** Western blot and immunofluorescence assays were used to measure the expression levels of epithelial protein markers (E-cadherin and β-catenin) and the expression levels of mesenchymal markers (N-cadherin and vimentin) in cells transfected with si-SNHG16. Error bars represent the mean ± SD of at least three independent experiments. ^*^p<0.05, ^**^p<0.01 vs. control group.

To evaluate the effect of SNHG16 on cell migration, transwell assay was employed. As demonstrated in Figure 3A, cells transfected with si-SNHG16 exerted a relative weaken migration capacity, compared with control cells. To investigate the underlying mechanism, we made further study. It has been revealed that EMT, a well-known feature of many carcinomas, is a critical process associated with a shift to aggressive cell migration characteristics. To determine whether EMT process is involved in the si-SNHG16-mediated weaken migration capacity of ESCC cells, we performed western blot and immunofluorescence assay to measure the expression levels of epithelial protein markers (E-cadherin and β-catenin) and the expression levels of mesenchymal markers (N-cadherin and vimentin) in cells transfected with si-SNHG16. As obtained in Figure 3B, results from western blot assay revealed that silencing SNHG16 could significantly reduce the level of mesenchymal markers but increase the level of epithelial markers. Likewise, immunofluorescence results showed that knockdown of SNHG16 could reverse the EMT phenotype to the MET (Figure 3C). All these investigations indicated that SNHG16 could promote ESCC cell migration through regulating the EMT process.

### MiR-140-5p was identified as a target of SNHG16

**Figure 4:**
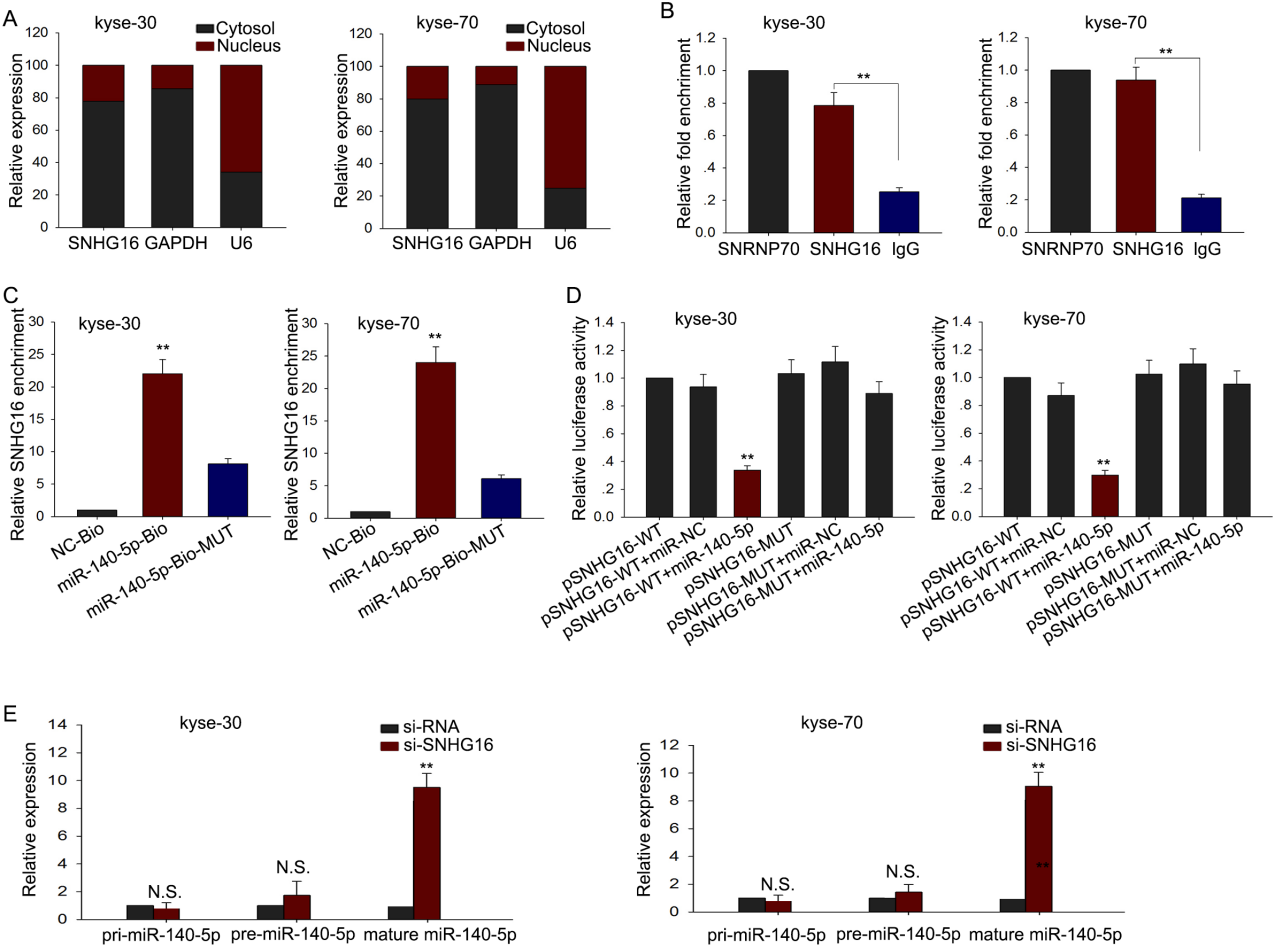
MiR-140-5p was identified as a target of SNHG16 **(A)** Cellular localization of SNHG16 was measured by qRT-PCR. **(B-D)** RIP, RNA pulldown and dual-luciferase assays were performed to investigate the interaction between SNHG16 and miR-140-5p. **(E)** The effect of SNHG16 knockdown on pri-miR-140-5p, pre-miR-140-5p and mature miR-140-5p was measured. Error bars represent the mean ± SD of at least three independent experiments. ^*^p<0.05, ^**^p<0.01 vs. control group.

Accumulating documents have been demonstrated that many lncRNAs are identified as ceRNAs for specific miRNAs. To investigate whether SNHG16 exerts its function in ESCC through acting as a ceRNA, we first measured the cellular localization of SNHG16. As observed in Figure 4A, the level of SNHG16 was mainly in cytoplasm, indicating that SNHG16 exerted its function at post-transcriptional level. Therefore, we hypothesized that SNHG16 might act as a ceRNA in our study. To confirm our hypothesis we utilized bioinformatics prediction software (http://starbase.sysu.edu.cn/mirLncRNA.php) to predict the miRNAs that may interact with SNHG16. Based on the bioinformatics analysis, we found that 64 miRNAs could form complementary base pairing with SNHG16 (Supplementary Table 1). We detected the expression levels of the 64 miRNAs in ESCC cells transfected with si-SNHG16, and found that only miR-140-5p was the most dramatically affected one (Supplementary Table 1). Therefore, we hypothesized that miR-140-5p was the downstream target of SNHG16 and chose miR-140-5p as the study object in the following experiments. To investigate the interaction between SNHG16 and miR-140-5p, we performed RIP, RNA pulldown and dual-luciferase report assay. As shown in Figure 4B, results from RIP showed that SNHG16 was detected in Ago2 immunoprecipitates from the control group but the level of SNHG16 were drastically reduced in Ago2 complexes purified from cells treated with miR-140-5p inhibitor, indicating that SNHG16 is likely in the miR-140-5p RISC complex. And results from RNA pulldown (Figure 4C) showed that SNHG16 was pulled down by miR-140-5p, while the bind site of SNHG16 for miR-140-5p was mutated failed to pull down SNHG16, suggesting that SNHG16 regulated miR-140-5p in a sequence-specific manner. Furthermore, the results of luciferase reporter assays provided further validation. As shown in Figure 4D, miR-140-5p mimics reduced the luciferase activity of wild-type (WT) SNHG16 reporter vector but not that of mutant reporter vector. Last, we examined the expression level of pri-miR-140-5p, pre-miR-140-5p and mature miR-140-5p in response to SNHG16 down-expressed. As shown in Figure 4E, no significant change was observed in pri-miR-140-5p and pri-miR-140-5p level, but the level of mature miR-140-5p was obviously increased. All these findings revealed that SNHG16 regulated miR-140-5p at post-transfection level through acting as a ceRNA.

### MiR-140-5p negatively regulated proliferation and migration of ESCC cells

**Figure 5:**
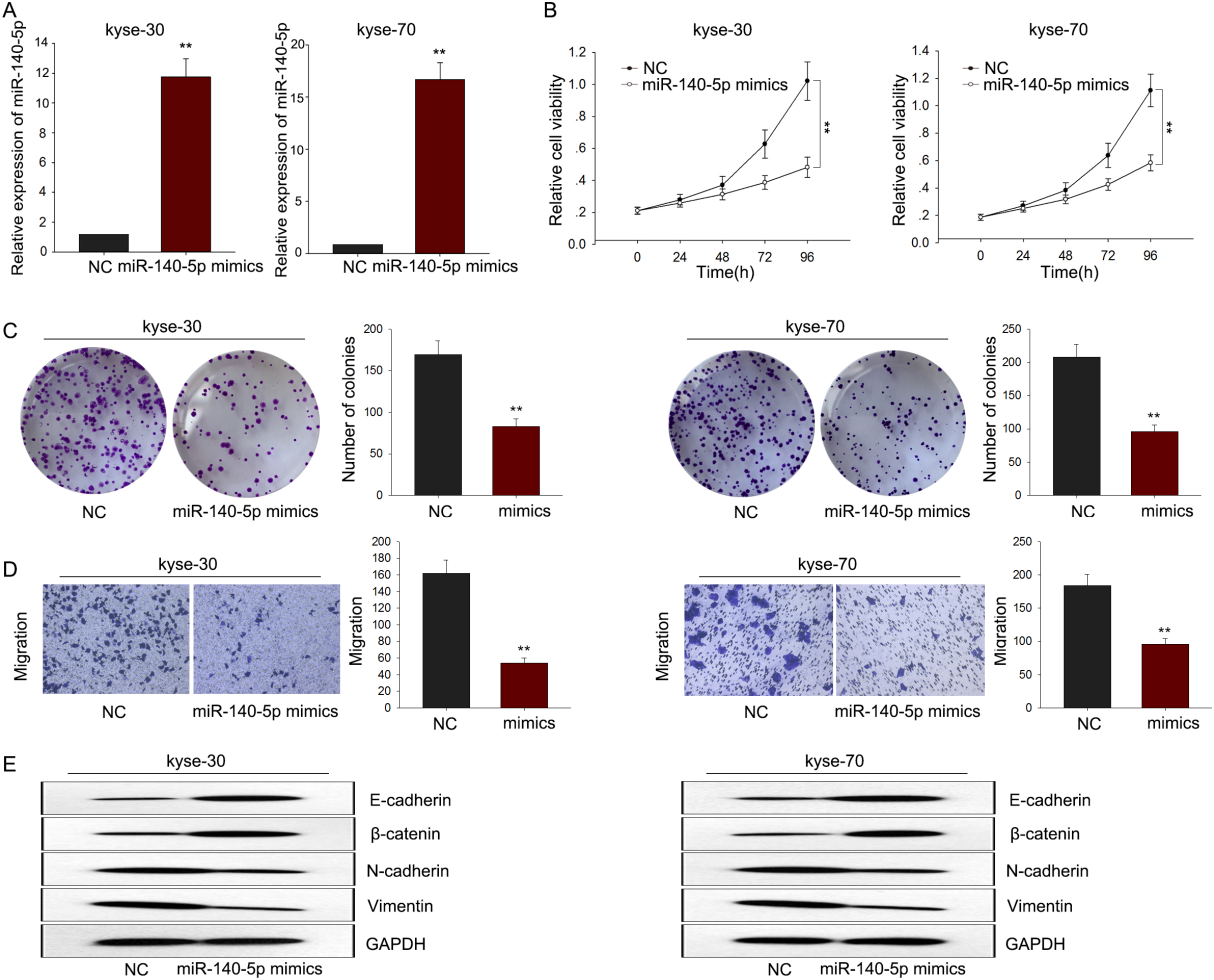
MiR-140-5p negatively regulated proliferation and migration of ESCC cells **(A)** qRT-PCR was performed to detect the transfection efficiency of miR-140-5p mimics. **(B-C)** MTT and colony formation assay was performed to detect the proliferation ability of ESCC cells transfected with miR-140-5p mimics. **(D)** Transwell assays were performed to determine the effect of miR-140-5p on cell migration ability. **(E)** Western blot assay revealed that overexpressed miR-140-5p could reverse the EMT phenotype to the MET of ESCC cells. Error bars represent the mean ± SD of at least three independent experiments. ^*^p<0.05, ^**^p<0.01 vs. control group.

To investigate the biological function of miR-140-5p in ESCC, miR-NC or miR-140-5p mimics were transfected into kyse-30 and kyse-70 cells. Satisfactory transfection efficiency was obtained after 48 h (Figure 5A). MTT and colony formation assays revealed weakened proliferation ability of kyse-30 and kyse-70 cells transfected with miR-140-5p mimics (Figure 5B–5C). Moreover, results from transwell assays revealed weakened metastasis capacity in kyse-30 and kyse-70 cells transfected with miR-140-5p mimics (Figure 5D). And western blot assay revealed that overexpressed miR-140-5p could reverse the EMT phenotype to the MET (Figure 5E). These data together suggested that miR-140-5p might be involved in the progression of ESCC.

### The oncogenic function of SNHG16 in ESCC cells was dependent on miR-140-5p

**Figure 6:**
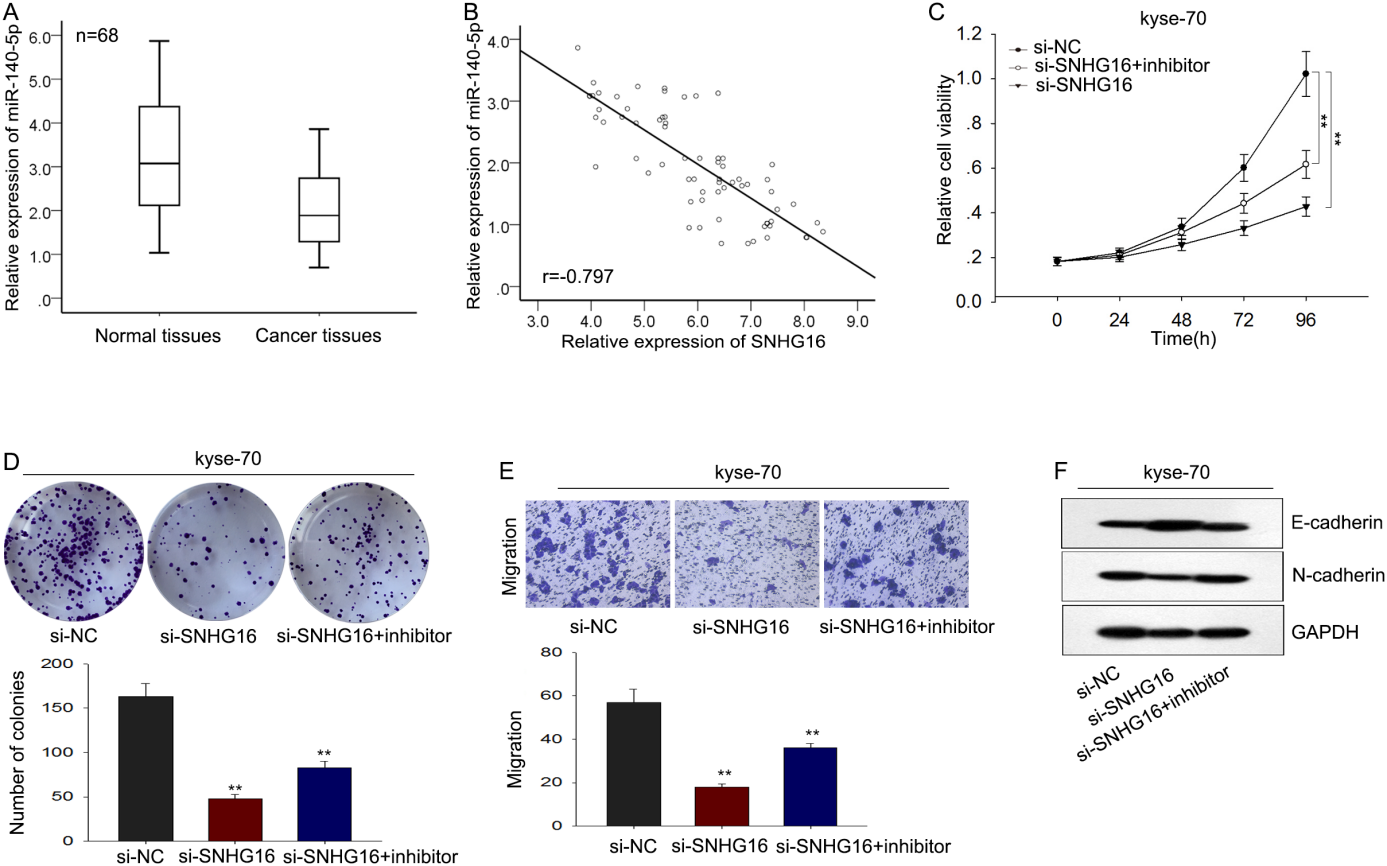
The oncogenic function of SNHG16 in ESCC cells was dependent on miR-140-5p **(A-B)** The level of miR-140-5p in ESCC tissues was measured and the expression correlation between miR-140-5p and SNHG16 in ESCC tissues was analyzed by 2-tailed Spearman’s correlation analysis. **(C-D)** MTT and colony formation assays were used to detect the proliferation ability of ESCC cells co-transfected with si-SNHG16 and miR-140-5p inhibitor. **(E-F)** Transwell and western blot assay were used to measure the migration capacity and EMT process of ESCC cells co-transfected with si-SNHG16 and miR-140-5p inhibitor. Error bars represent the mean ± SD of at least three independent experiments. ^*^p<0.05, ^**^p<0.01 vs. control group.

To further validate the relationship between miR-140-5p and SNHG16 in ESCC, we measured the level of miR-140-5p in ESCC tissues and analyzed the expression correlation between miR-140-5p and SNHG16 in ESCC tissues. As shown in Figure 6A-6B, the level of miR-140-4p in ESCC tissues was significantly lower than that in corresponding normal tissues, which was negatively correlated with the level of SNHG16 (2-tailed Spearman’s correlation, r=-0.797, p<0.01). Afterwards, rescue experiments were applied to determine whether SNHG16 influenced ESCC cells proliferation and migration in a miR-140-5p-dependent manner. MiR-NC or miR-140-5p inhibitor was transfected into kyse-70 cells transfected with SNHG16. MTT and colony formation assays showed that the weakened proliferation induced by si-SNHG16 in kyse-70 cells was in part abrogated by the introduction of miR-140-5p inhibitor (Figure 6C-6D). Likewise, transwell and western blot assay revealed that a decreased migration capacity and EMT process block caused by si-SNHG16 was partially abolished by miR-140-5p inhibitor in kyse-70 cells (Figure 6E-6F). These data indicated that SNHG16 contributed to the progression of ESCC cells through targeting miR-140-5p.

### SNHG16 positively regulated the miR-140-5p target gene ZEB1 in ESCC

**Figure 7:**
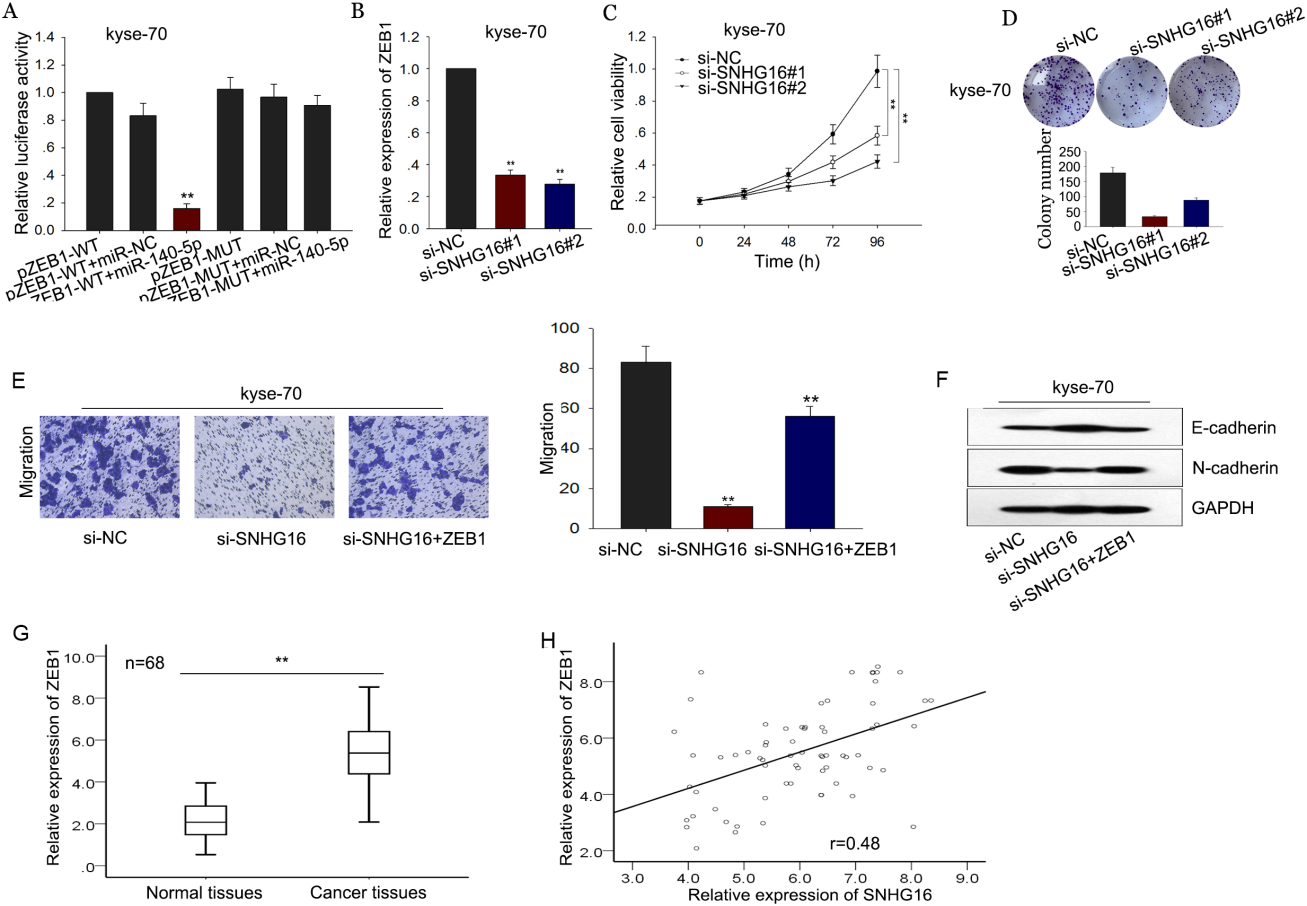
SNHG16 positively regulated the miR-140-5p target gene ZEB1 in ESCC **(A)** Luciferase assays were performed to measure the interaction between miR-140-5p and ZEB1. **(B)** The level of ZEB1 was detected in response to SNHG16 knockdown by qRT-PCR. **(C-D)** MTT and colony formation assays were used to detect the proliferation ability of ESCC cells co-transfected with si-SNHG16 and ZEB1. **(E-F)** Transwell and western blot assay were used to measure the migration capacity and EMT process of ESCC cells co-transfected with si-SNHG16 and ZEB1. **(G-H)** The level of ZEB1 in ESCC tissues was measured and the expression correlation between ZEB1 and SNHG16 in ESCC tissues was analyzed by 2-tailed Spearman’s correlation analysis. Error bars represent the mean ± SD of at least three independent experiments. ^*^p<0.05, ^**^p<0.01 vs. control group.

Based on the microRNA.org, we found that ZEB1 is a potential target of miR-140-5p. ZEB1 had been reported to promote EMT in multiple tumors, including ESCC [25–27]. Therefore, it is reasonable to speculte that ZEB1 is involved in the SNGH16/miR-140-5p-mediated EMT formation. We performed luciferase assays and found that ZEB1 was a target of miR-140-5p (Figure 7A). Then, we measured the level of ZEB1 in response to SNHG16 knockdown, as shown in Figure 7B, we observed that down expression of SNHG16 decreased the level of ZEB1, indicating that ZEB1 is involved in the function exerted by SNHG16. Thus, we proposed that SNHG16 and ZEB1 interacted with miR-140-5p by functioning as ceRNAs. Herein, to confirm such model, rescue experiments were performed. ZEB1 expression vector or empty vector was transfected into kyse-70 cells transfected with si-SNHG16. MTT and colony formation assays showed that the weakened proliferation induced by si-SNHG16 in kyse-70 cells was partially rescued by the introduction of ZEB1, (Figure 7C-7D). Additionally, transwell assay revealed that decreased migration capacity caused by si-SNHG16 was in part reversed by ZEB1 in kyse-70 cells (Figure 7E). And western blot assay revealed that si-SNHG16-mediated MET could be reversed by ZEB1 introduction (Figure 7F). Additionally, the expression of ZEB1 in ESCC tissues and corresponding non-carcinoma tissues was determined by qRT-PCR. The relative levels of ZEB1 in ESCC tissues were significantly increased compared to the corresponding normal tissues (Figure 7G). Additionally, the level of ZEB1 showed a positive correlation with SNHG16 level (2-tailed Spearman’s correlation, r=0.48, p<0.01; Figure 7H).

### SNHG16 regulated the proliferation of ESCC cells *in vivo*

**Figure 8:**
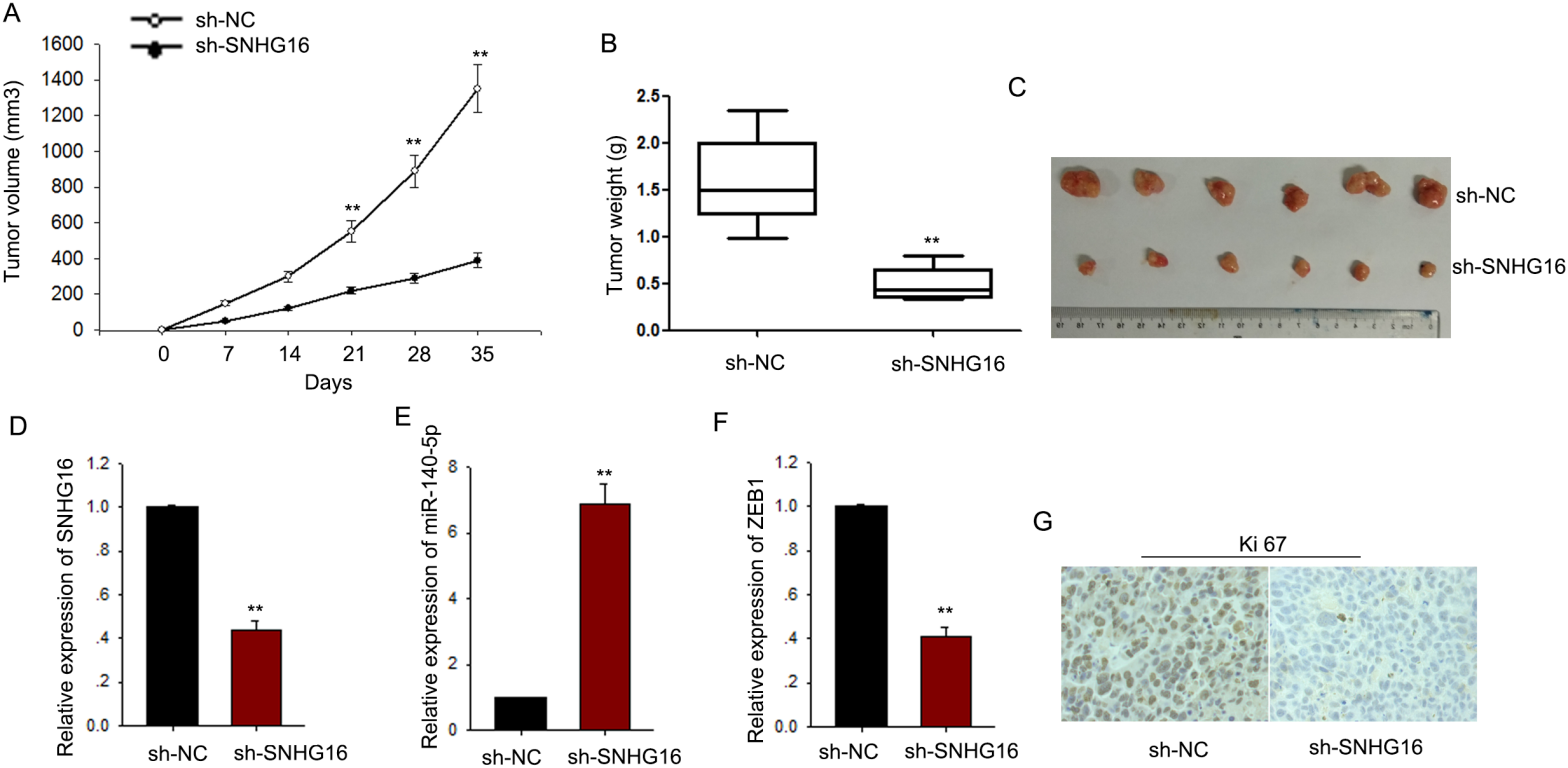
SNHG16 regulated the proliferation of ESCC cells *in vivo* **(A-C)** The tumor volume, weight and morphology were present. **(D-F)** The levels of SNHG16, miR-140-5p and ZEB1 in tumor tissues were measured by qRT-PCR. **(G)** Immunostaining analysis was performed to measure the positive rate of Ki67 in tumors derived from sh-SNHG16 transfected kyse-70 cells. Error bars represent the mean ± SD of at least three independent experiments. ^*^p<0.05, ^**^p<0.01 vs. control group.

**Figure 9:**
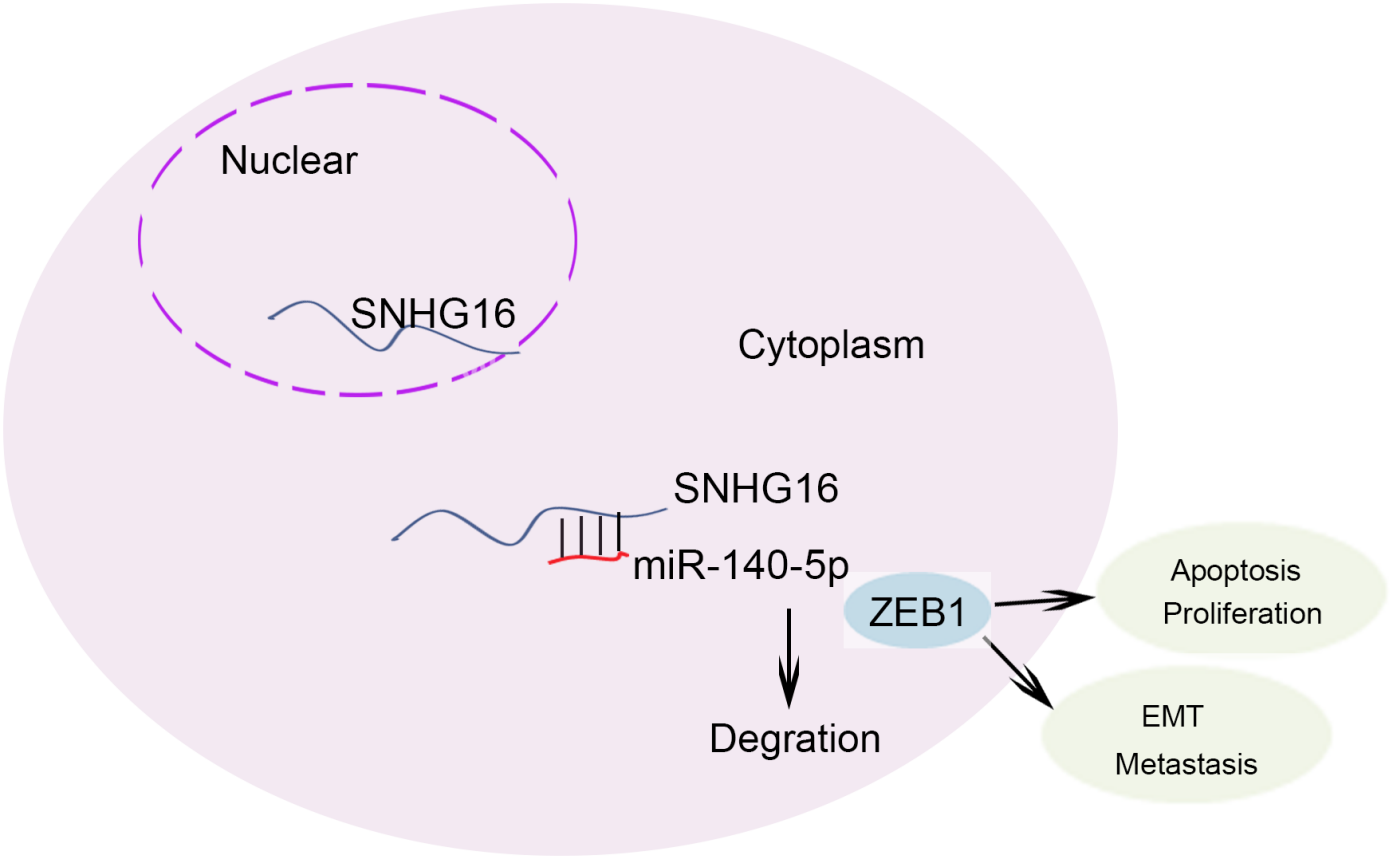
The regulation model description of SNHG16/miR-140-5p/ZEB1 signal pathway

To assess the effects of SNHG16 on ESCC cell proliferation *in vivo*, we inoculated nude mice with kyse-70 cells stably transfected with SNHG16 shRNA. Tumors derived from sh-SNHG16 transfected kyse-70 cells grew more slowly than those derived from control shRNA transfected cells (Figure 8A-8C). qRT-PCR showed that the level of SNGH16 and ZEB1 in the tumor tissues derived from sh-SNHG16 transfected kyse-70 cells was significantly decreased, while the level of miR-140-5p was obviously increased (Figure 8D-8F). Immunostaining analysis revealed a lower positive rate of Ki67 in tumors derived from sh-SNHG16 transfected kyse-70 cells compared with the control groups (Figure 8G). Collectively, these data indicated that there is a regulatory signaling pathway in which SNHG16 regulated ZEB1 by competitively sponging miR-140-5p (Figure 9), inducing increased proliferation ability and enhanced migration capacity in ESCC cells.

## DISCUSSION

LncRNAs have been proposed as critical regulators in biological processes and human diseases through modulating gene expression programs at transcriptional, post-transcriptional and epigenetic level. For example, Xia et al. revealed that papillary thyroid carcinoma susceptibility candidate 3 (PTCSC3) inhibits proliferation and invasion of glioma cells by suppressing the Wnt/beta-catenin signaling pathway [28]; Zhang et al. demonstrated that H3K27 acetylation activated-long non-coding RNA CCAT1 affects cell proliferation and migration by regulating SPRY4 and HOXB13 expression in esophageal squamous cell carcinoma [29]; and Austin et al. reported that l Transcriptional profiling identifies the long noncoding RNA plasmacytoma variant translocation (PVT1) as a novel regulator of the asthmatic phenotype in human airway smooth muscle [30]. Targeting lncRNAs-based signal pathway might be novel therapy methods. However, the roles of lncRNAs in ESCC carcinogenesis are not well explored. Deeply investigating the molecular mechanism underlying the initation and progression of ESCC is essential for facilitating the exploitation of novel therapeutic targets.

SNHG16 has been identified as an oncogene in many cancers; however, its function in ESCC is not completely investigated. In the present study, based on the results RT–qPCR, we demonstrated that the SNHG16 is upregulated in ESCC tissues and acts as an oncogene in tumorigenesis. And the level of SNHG16 expression was strikingly higher in ESCC cell lines. Applying loss-of-function approaches, we identified that SNHG16 plays an oncogenic role in ESCC cell proliferation, migration and EMT formation. Silenced SNHG16 significantly decreased ESCC cell growth ability, reduced cell migration capacity and inhibited the EMT phenotype formation. Our findings revealed that SNHG16 may function as an oncogene in ESCC. However, the underlying mechanism by which SNHG16 functions in ESCC warrants to be further investigated.

MicroRNAs play significant roles in the progression of various diseases, including tumorigenesis. And currently, many miRNAs are identified as disease-related [31–35]. It has been proposed that endogenous transcripts containing miRNA response elements (MREs) can interact with each other by acting as miRNA sponges or ceRNAs, forming large-scale regulatory networks across the transcriptome [15, 16]. For example, Tan et al. demonstrated that double-negative feedback loop between long non-coding RNA TUG1 and miR-145 promotes epithelial to mesenchymal transition and radioresistance in human bladder cancer cells [36]; and Ji et al. long non-coding RNA TUG1 on gastric cancer cell transference and invasion through regulating and controlling the expression of miR-144/c-Met axis [37]. In our study, we provide evidence that SNHG16 may function as a ceRNA by competitively sponging miR-140-5p and influence the distribution on target gene ZEB1. Consistent with the effect of miR-140-5p mimic, silenced SNHG16 could suppress miR-140-5p target ZEB1. Therefore, the influence of SNHG16 on ESCC cells proliferation, migration and EMT formation could be owned to function as a ceRNA competitively sponging miR-140-5p.

Collectively, we demonstrated that the SNHG16 is upregulated in ESCC tissues and cell lines. Its effects on cell proliferation, migration and EMT formation hint its oncogenic role in ESCC tumorigenesis. In our study, SNHG16 acts as a molecular sponge for miR-140-5p and regulates its target ZEB1. This interaction relationship of SNHG16 and miR-140-5p may highlight the important role of RNA–RNA interaction and provide novel insight into lncRNA-based mechanisms underlying various aspects of tumorigenesis.

## MATERIALS AND METHODS

### Patients and specimens

68 carcinogenic tissues and corresponding normal tissues were selected from patients with ESCC who underwent esophagectomy at Jinling Hospital of Nanjing from April 2007 to April 2016. All patients were pathologically diagnosed with ESCC and were not subject to preoperative chemotherapy and/or radiotherapy. Tissue specimens were obtained with the consent of all patients. Tissues were stored in liquid nitrogen until RNA was extracted. ESCC diagnosis was defined according to the tumor node metastasis stage classification and World Health Organization (WHO) criteria. This study was approved by the ethics committee of Jiangsu Province Medical Association.

### Cell cultures

The ESCC cell lines eca109, EC9706, TE1, Kyse-30 and Kyse-70 and the normal esophageal epithelial cell line HEEC were purchased from Fudan University Shanghai Cell Bank and were cultured at 37° C in a humidified incubator 5% CO_2_. Cells were incubated in RPMI 1640 (Gibco, Carlsbad, USA) supplemented with 10% fetal bovine serum (FBS; Gibco), 100 U/ml penicillin and 100 μg/ml streptomycin.

### Cell transfection

Hsa-miRNA-140-5p mimic/negative control mimic and hsa-miRNA-140-5p inhibitor/negative control inhibitor were purchased from Applied Biological Materials (GenePharma, Shanghai, China). The siRNA specifically targeting SNHG16 were synthesized by GenePharma. The siRNA sequence for SNHG16 was si-SNHG16-1, 5’-CATGTCCTTCTGATCACCAAGTTGACTTA-3’, si-SNHG16-2, 5’-GATATCTTAGTCCTAACCATATTGATCCC-3’. Transfections were performed using the Lipofectamine 2000 kit (Invitrogen) according to the manufacturer’s instructions. Cell lines stably suppressing SNHG16 were constructed by transfecting with sh-RNA construct containing desired vector, and screened with G418 (2μg/ml) for three months.

### Real-time quantitative reverse-transcription polymerase chain reaction (qRT-PCR)

Total RNA from tissues and cells was isolated with Trizol reagent (Invitrogen, CA, USA) according to the manufacturer’s instructions. Reverse transcription was performed with PrimeScript RT reagent Kit (Takara, Japan) according to the manufacturer’s protocol. qRT-PCR was performed with SYBR Prime Script RT-PCR Kits (Takara, Japan) based on the manufacturer’s instructions. The miR-140-5p, SNHG16 and ZEB1 level was calculated with the 2^-ΔΔCt^ method, which was normalized to u6 and GAPDH mRNA, respectively. All assays were performed in triplicate. The expression levels were relative to the fold change of the corresponding controls, which were defined as 1.0.

### Cell viability

Cell viability was assessed via 3-(4,5-dimethylthiazol-2-yl)-2, 5-diphenyl-trtrazolium bromide (MTT) assay. 5 × 10^3^ cells/well were seeded in a 96-well flat-bottomed plate for 24 h, then transfected with corresponding si-RNA and cultured in normal medium. At 0, 24, 48, 72 h and 96h after transfection, the MTT solution (5 mg/ml, 20 μl) was added to each well. Following incubation for 4 h, the media was removed and 100 μl DMSO were added to each well. The relative number of surviving cells was assessed by measuring the optical density (O.D.) of cell lysates at 560 nm. All assays were performed in triplicate.

### Colony formation assay

Cells (500 cells/well) were plated in 6-well plates and incubated in DMEM with 10% bovine calf serum at 37 °C. Two weeks later, the cells were fixed and stained with 0.1% crystal violet. The number of visible colonies was counted manually.

### Flow cytometric analysis of apoptosis

Cells transfected with indicated plasmid or negative control were reaped after 48 hours. Apoptosis was performed using flow cytometric analyses with Annexin V: FITC Apoptosis Detection Kits (BD Biosciences, USA), according to the manufacturer’s instructions. All samples were assayed in triplicate.

### Cell migration

Cell migration was measured by transwell chamber (8 um pore size, Corning). 48 h after transfection, cells in serum-free media were placed into the upper chamber. Media containing 10% bovine calf serum was added into the lower chamber. Following 48 h incubation, cells remaining in upper membrane were wiped off, while cells that migrated were fixed in methanol, stained with 0.1% crystal violet and counted under a microscope. Three independent experiments were carried out.

### Dual luciferase reporter assay

PmirGLO-SNHG16-wt or pmirGLO-SNHG16-mut (miR-140-5p) was co-transfected with miR-140-5p mimics or miR-NC into HEK293 cells by Lipofectamine-mediated gene transfer. The relative luciferase activity was normalized to Renilla luciferase activity 48h after transfection. The data were relative to the fold change of the corresponding control groups defined as 1.0.

### RNA immunoprecipitation (RIP)

RNA immunoprecipitation was performed using thermo fisher RIP kit (Thermo, USA) based on the manufacturer’s protocol. The Ago2 antibodies are purchased from Abcam (USA). Normal mouse IgG (Abcam, USA) was applied as negative control and anti-SNRNP70 (Abcam, USA) was employed as positive control for the RIP procedure. Purified RNA was subjected to qRT-PCR analysis to demonstrate the presence of the binding targets using respective primers.

### RNA-pull down assay

LncRNA-SNHG16 transcripts were transcribed using T7 RNA polymerase (Ambio life) *in vitro*, then by using the RNeasy Plus Mini Kit (Qiagen) and treated with DNase I (Qiagen). Purified RNAs were biotin-labeled with the Biotin RNA Labeling Mix (Ambio life). Then, magnetic beads were added to each binding reaction, and incubated at room temperature. Finally, the beads were washed, and the eluted proteins were detected by western blot analysis.

### Cell cytoplasm/nucleus fraction isolation

Pairs Kit (Thermo Scientific, Waltham, MA, USA) was employed to prepare cytoplasmic and nuclear extracts from MG-63 cells. In detail, Collect up to 10^7^ fresh cultured ESCC cells, wash once in PBS, and place washed cells on ice; resuspend cells in 100–500 μL ice-cold cell fractionation buffer; incubate on ice 5–10 min; centrifuge samples 1–5 min at 4°C and 500 xg; carefully aspirate the cytoplasmic fraction away from the nuclear pellet and wash the nuclear pellet in ice-cold cell fractionation buffer; then lyse nuclear pellet in cell disruption buffer and split the sample for RNA isolation. For RNA isolation, mix the lysate with an equal volume of 2X Lysis/Binding Solution; add 1 “sample volume” of 100% ethanol to the mixture; wash with wash solution; elute RNA with elute solution. RNAs extracted from each of the fractions were subjected to following RT–qPCR analysis to demonstrate the levels of nuclear control transcript (U6), cytoplasmic control transcript (GAPDH).

### Western blot assay

All antibodies (E-cadherin (1: 2000 dilution), β-catenin (1: 2000 dilution) N-cadherin (1: 2000 dilution), vimentin (1: 2000 dilution), ZEB1 (1: 2000 dilution), GAPDH (1:3000 dilution) were purchased from Abcam (USA). Membranes were blocked with 5% (v/v) milk and incubated with the primary antibodies in 5% (w/v) bovine serum albumin (BSA) at 4°C overnight, followed by incubation with the corresponding horseradish peroxidase-linked secondary antibodies. Blots were washed for 15 min three times and the signals were detected using an enhanced chemiluminescence-detecting kit (Thermo Fisher, MA, USA) followed by exposure with Tanon 5200 Biotanon, China).

### Immunofluorescence

Cells seeded on glass coverslips in 6-well plates were fixed in 4% formaldehyde solution and permeabilized with 0.5% Triton X-100/PBS. Cells were blocked with 5% BSA-PBS for 1h at room temperature and incubated with primary antibody at 4°C overnight, followed by incubation with fluorescent-dye conjugated secondary antibody (Invitrogen) for 1h, and then stained with DAPI. Finally, images were taken under an inverted fluorescence microscope.

### Xenograft transplantation and immunohistochemistry

Approximately 5.0^*^10^6^ kyse-70 cells suspended in 100 μl PBS and stably transfected with shRNA/SNHG16 or shRNA/control were injected subcutaneously into the right side of the posterior flank of female BALB/c athymic nude mice (Department of Comparative Medicine, Jinling Hospital) at five to six weeks of age. Tumor growth was examined every other day with a vernier caliper. Tumor volumes were calculated by using the equation: V=A^*^B^2^/2 (mm^3^), where A is the largest diameter and B is the perpendicular diameter. After five weeks, all mice were killed and necropsies were carried out. The primary tumors were excised, paraffin-embedded, formalin-fixed, and conducted immunostaining analysis for Ki-67 protein expression according to the manufacturer's instructions.

### Statistical analysis

Data were shown as the means ± standard error of at least three independent experiments. The SPSS 17.0 software (SPSS Inc., Chicago, IL, USA) was used for statistical analysis. Two group comparisons were performed with a Student t test. Multiple group comparisons were analyzed with one-way ANOVA. Statistically significant positive correlation between SNHG16 and miR-140-5p or ZEB1 levels in ESCC tissues was analyzed by Spearman’s correlation analysis. All tests performed were two-sided. P < 0.05 was considered statistically significant.

## SUPPLEMENTARY MATERIALS TABLE




