# Supplementary material for: SNHG16/miR-140-5p axis promotes esophagus cancer cell proliferation, migration and EMT formation through regulating ZEB1
Source: Oncotarget. 2017 Dec 11;9(1):1028–40. doi: 10.18632/oncotarget.23178 (PMC5787416; doi:10.18632/oncotarget.23178)
Supplement: Supplementary file 1 [file oncotarget-09-1028-s001.pdf]

SNHG16/miR-140-5p axis promotes esophagus cancer cell proliferation, migration and EMT formation through regulating ZEB1

SUPPLEMENTARY MATERIALS

Supplementary Table 1: Initial profiling of miRNAs

See Supplementary File 1
